# Supplementary material for: Characterization of Two Mitogenomes of Hyla sanchiangensis (Anura: Hylidae), with Phylogenetic Relationships and Selection Pressure Analyses of Hylidae
Source: Animals (Basel). 2023 May 10;13(10):1593. doi: 10.3390/ani13101593 (PMC10215353; doi:10.3390/ani13101593)
Supplement: Supplementary file 1 [file animals-13-01593-s001.zip › Table S1 The third codon saturation.pdf]

Table S1. The third codon saturation results are Iss< iss.csym,Iss<Iss. cAsym, $p < 0.05$, indicating that the third codon is unsaturated, the results are credible.

NumOTU	Iss	Iss.cSym	T	DF	P	Iss.cAsym	T	DF	P
4	0.306	0.857	107.177	10547	0.0000	0.846	105.003	10547	0.0000
8	0.305	0.845	94.903	10547	0.0000	0.762	80.417	10547	0.0000
16	0.302	0.849	92.609	10547	0.0000	0.676	63.348	10547	0.0000
32	0.305	0.817	84.822	10547	0.0000	0.571	44.126	10547	0.0000
