# Supplementary material for: Characterization of Two Mitogenomes of Hyla sanchiangensis (Anura: Hylidae), with Phylogenetic Relationships and Selection Pressure Analyses of Hylidae
Source: Animals (Basel). 2023 May 10;13(10):1593. doi: 10.3390/ani13101593 (PMC10215353; doi:10.3390/ani13101593)
Supplement: Supplementary file 1 [file animals-13-01593-s001.zip › Table S2 RSCU.pdf]

Table S2. The codon number and relative synonymous codon usage in mitochondrial protein-coding genes.

Codon	Count		RSCU		Codon	Count		RSCU		Codon	Count		RSCU		Codon	Count		RSCU	
	WC	JX	WC	JX		WC	JX	WC	JX		WC	JX	WC	JX		WC	JX	WC	JX
UUU(F)	160	151	1.32	1.24	UCU(S)	73	75	1.49	1.55	UAU(Y)	54	54	0.94	0.94	UGU(C)	13	14	0.84	0.85
UUC(F)	83	92	0.68	0.76	UCC(S)	61	62	1.24	1.28	UAC(Y)	61	61	1.06	1.06	UGC(C)	18	19	1.16	1.15
UUA(L)	158	163	1.61	1.65	UCA(S)	104	97	2.12	2	UAA(*)	0	0	0	0	UGA(W)	94	96	1.74	1.76
UUG(L)	37	28	0.38	0.28	UCG(S)	10	11	0.2	0.23	UAG(*)	0	0	0	0	UGG(W)	14	13	0.26	0.24
CUU(L)	124	123	1.26	1.24	CCU(P)	37	47	0.78	0.97	CAU(H)	34	36	0.74	0.78	CGU(R)	11	6	0.64	0.35
CUC(L)	85	85	0.86	0.86	CCC(P)	68	60	1.43	1.24	CAC(H)	58	56	1.26	1.22	CGC(R)	9	12	0.52	0.7
CUA(L)	148	164	1.51	1.66	CCA(P)	74	78	1.56	1.62	CAA(Q)	76	79	1.73	1.8	CGA(R)	41	45	2.38	2.61
CUG(L)	38	31	0.39	0.31	CCG(P)	11	8	0.23	0.17	CAG(Q)	12	9	0.27	0.2	CGG(R)	8	6	0.46	0.35
AUU(I)	217	219	1.35	1.34	ACU(T)	84	78	1.21	1.16	AAU(N)	65	69	0.98	1.08	AGU(S)	16	19	0.33	0.39
AUC(I)	105	107	0.65	0.66	ACC(T)	80	79	1.16	1.18	AAC(N)	67	59	1.02	0.92	AGC(S)	30	27	0.61	0.56
AUA(M)	114	119	1.43	1.5	ACA(T)	104	104	1.5	1.55	AAA(K)	82	79	1.89	1.82	AGA(*)	0	0	0	0
AUG(M)	46	40	0.57	0.5	ACG(T)	9	7	0.13	0.1	AAG(K)	5	8	0.11	0.18	AGG(*)	0	0	0	0
GUU(V)	58	62	1.3	1.39	GCU(A)	75	76	0.97	0.98	GAU(D)	33	29	0.92	0.77	GGU(G)	36	39	0.66	0.72
GUC(V)	39	39	0.87	0.87	GCC(A)	128	124	1.65	1.6	GAC(D)	39	46	1.08	1.23	GGC(G)	57	52	1.05	0.96
GUA(V)	60	54	1.34	1.21	GCA(A)	101	103	1.3	1.33	GAA(E)	75	71	1.76	1.65	GGA(G)	84	82	1.55	1.52
GUG(V)	22	24	0.49	0.54	GCG(A)	6	7	0.08	0.09	GAG(E)	10	15	0.24	0.35	GGG(G)	40	43	0.74	0.8
