# Supplementary material for: Characterization of Two Mitogenomes of Hyla sanchiangensis (Anura: Hylidae), with Phylogenetic Relationships and Selection Pressure Analyses of Hylidae
Source: Animals (Basel). 2023 May 10;13(10):1593. doi: 10.3390/ani13101593 (PMC10215353; doi:10.3390/ani13101593)
Supplement: Supplementary file 1 [file animals-13-01593-s001.zip › Table S3. The best partitioning scheme and best-fitting models are obtained by using PartitionFinder program.pdf]

Table S3. The best partitioning scheme and best-fitting models are obtained by using PartitionFinder program. The full names of all abbreviations are as follows: pos1: first codon; pos2: second codon; pos2: thrid codon; GTR: general time reversible; I: unchanged site proportion; G: Gamma distribution.

Nucleotide Sequence Alignments		
**Subset**	**Subset Partitions**	**Best Model**
Partition 1	ATP8_pos1,ND2_pos1, ND4L_pos1, ATP6_pos1, ND5_pos1, ND4_pos1, ND3_pos1, ND1_pos1	GTR+I+G
Partition 2	ND1_pos2, ND4L_pos2, ATP6_pos2, ATP8_pos2, ND3_pos2, ND2_pos2, ND4_pos2,ND5_pos2	TVM+I+G
Partition 3	ND4_pos3, ND3_pos3, ND4L_pos3, ND2_pos3, ATP6_pos3, ND1_pos3, ND5_pos3, ATP8_pos3	TRN+I+G
Partition 4	COI_pos1, COII_pos1, COIII_pos1, CYTB_pos1	SYM+I+G
Partition 5	COI_pos2, COII_pos2, COIII_pos2, CYTB_pos2	K81UF+I+G
Partition 6	COI_pos3	TRN+G
Partition 7	CYTB_pos3, COIII_pos3, COII_pos3	TIM+I+G
Partition 8	ND6_pos1, ND6_pos2	K81UF+G
Partition 9	ND6_pos3	TRN+G
