# Supplementary material for: Characterization of Two Mitogenomes of Hyla sanchiangensis (Anura: Hylidae), with Phylogenetic Relationships and Selection Pressure Analyses of Hylidae
Source: Animals (Basel). 2023 May 10;13(10):1593. doi: 10.3390/ani13101593 (PMC10215353; doi:10.3390/ani13101593)
Supplement: Supplementary file 1 [file animals-13-01593-s001.zip › Table S4 the Sequence ReadArchive (SRA) of 13 species for calculating the genetic distance.pdf]

Table S4. Sequence Read Archive (SRA) of 13 species for calculating the genetic distance.

Species	Voucher ID (alias)	SRA
Dryophytes suweonensis	mms6883_HYLSU	ERS4294464
Dryophytes suweonensis	mms6884_HYLSU	ERS4294465
Dryophytes suweonensis	mms6885_HYLSU	ERS4294466
Dryophytes suweonensis	mms4973_HYLSU	ERS4294468
Dryophytes suweonensis	mms4974_HYLSU	ERS4294469
Dryophytes suweonensis	mms5027_HYLSU	ERS4294470
Dryophytes immaculatus	mms8665_HYLIM	ERS4294472
Dryophytes immaculatus	mms8667_HYLIM	ERS4294474
Dryophytes flaviventris sp. nov.	mms8548_HYLFL	ERS4294476
Dryophytes flaviventris sp. nov.	mms8549_HYLFL	ERS4294477
Dryophytes flaviventris sp. nov.	mms8550_HYLFL	ERS4294478
Dryophytes flaviventris sp. nov.	mms8552_HYLFL	ERS4294480
Dryophytes flaviventris sp. nov.	mms8553_HYLFL	ERS4294481
