# Supplementary material for: Characterization of Two Mitogenomes of Hyla sanchiangensis (Anura: Hylidae), with Phylogenetic Relationships and Selection Pressure Analyses of Hylidae
Source: Animals (Basel). 2023 May 10;13(10):1593. doi: 10.3390/ani13101593 (PMC10215353; doi:10.3390/ani13101593)
Supplement: Supplementary file 1 [file animals-13-01593-s001.zip › Table S8 other models.doc]

| Model | np | Ln L | Estimates of parameters | | | Model compared | LRT P-value | Omega for Foreground Branch |
| --- | --- | --- | --- | --- | --- | --- | --- | --- |
| Two ratio Model 2 | 81 | -172012.812483 | ω: | ω0=0.05699 | ω1=0.06775 | Model 0 vs. Two ratio Model 2 | 0.208490390 | [] |
| Model 0 | 80 | -172013.603422 | ω= | 0.05711 | |  |  |  |

| Model | np | LnL | Site class 0 | | Site class 1 | | Site class 2 | | | | | | | Model compared | LRT P-value |
| --- | --- | --- | --- | --- | --- | --- | --- | --- | --- | --- | --- | --- | --- | --- | --- |
|  |  |  | ω0 | p0 | ω1 | p1 | wb | w2f1 | w2f2 | w2f3 | w2f4 | w2f5 | p2 | M2a_rel vs CmC | 0.153812691 |
| CmC | 84 | -162229.724579 | 0.00906 | 0.70836 | 1.00000 | 0.0161 | 0.15792 | 0.11829 |  |  |  |  | 0.27554 |  |  |
| M2a_rel | 83 | -162230.741592 | 0.00906 | 0.70845 | 1.00000 | 0.0161 | 0.15760 |  |  |  |  |  | 0.27546 |  |  |

Adaptive analysis of Branch model (BM)

Adaptive analysis of Clade model (CM)
