# Supplementary figures and images for: Characterization of Two Mitogenomes of Hyla sanchiangensis (Anura: Hylidae), with Phylogenetic Relationships and Selection Pressure Analyses of Hylidae
Source: Animals (Basel). 2023 May 10;13(10):1593. doi: 10.3390/ani13101593 (PMC10215353; doi:10.3390/ani13101593)

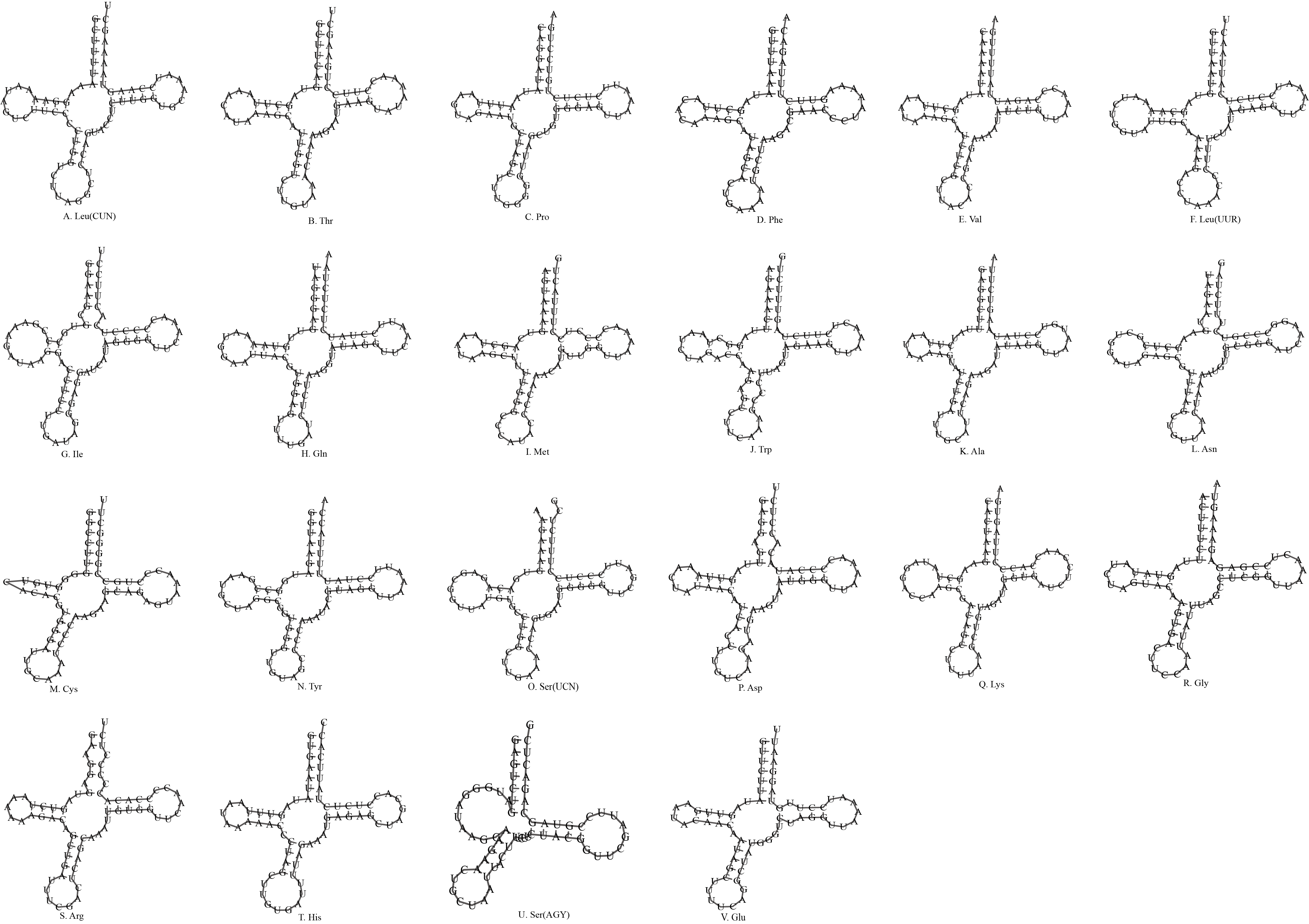

Supplement: Supplementary file 1 [file animals-13-01593-s001.zip › Figure S1 tRNA-WC.png]

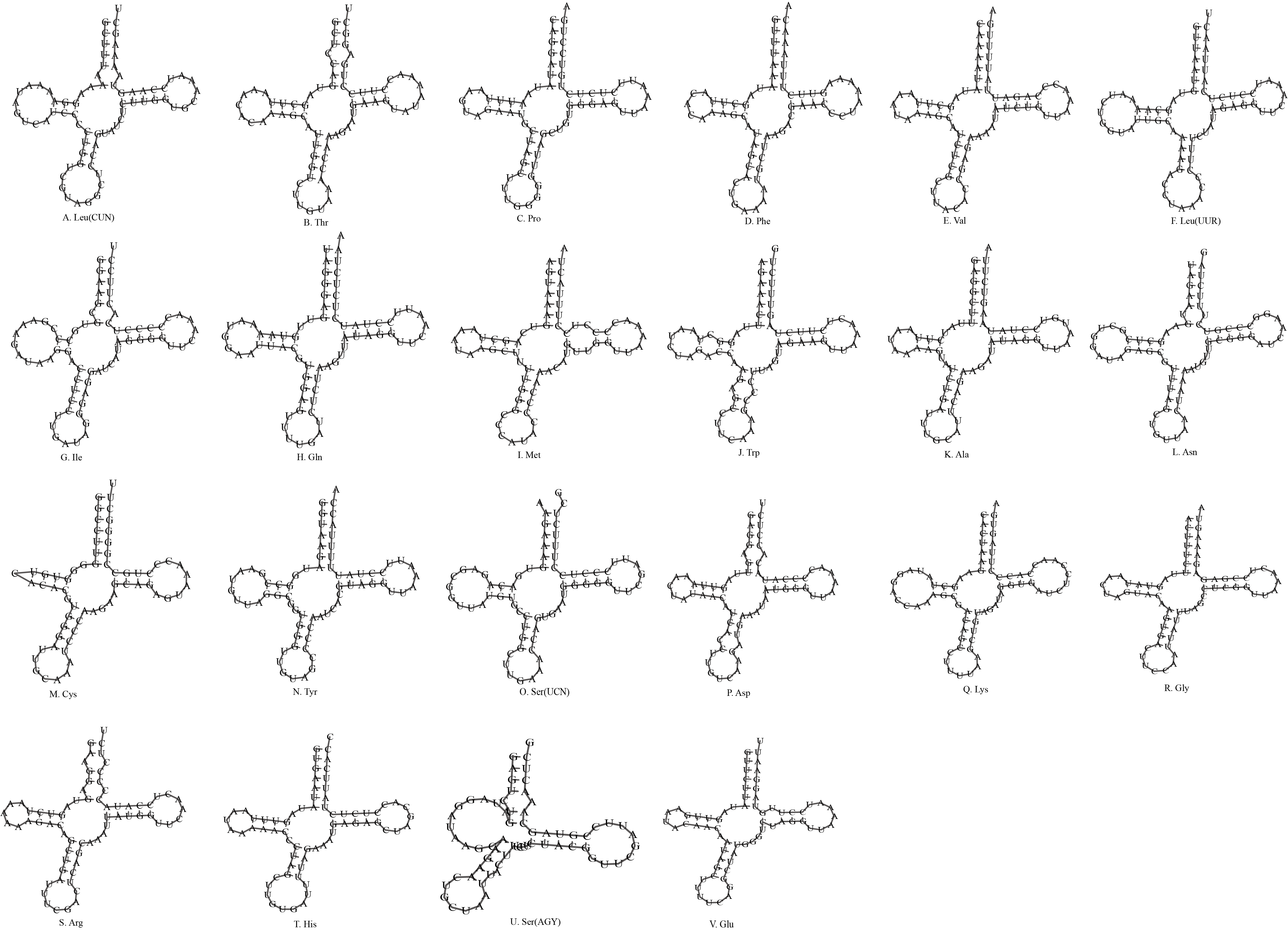

Supplement: Supplementary file 1 [file animals-13-01593-s001.zip › Figure S2 tRNA-JX.png]

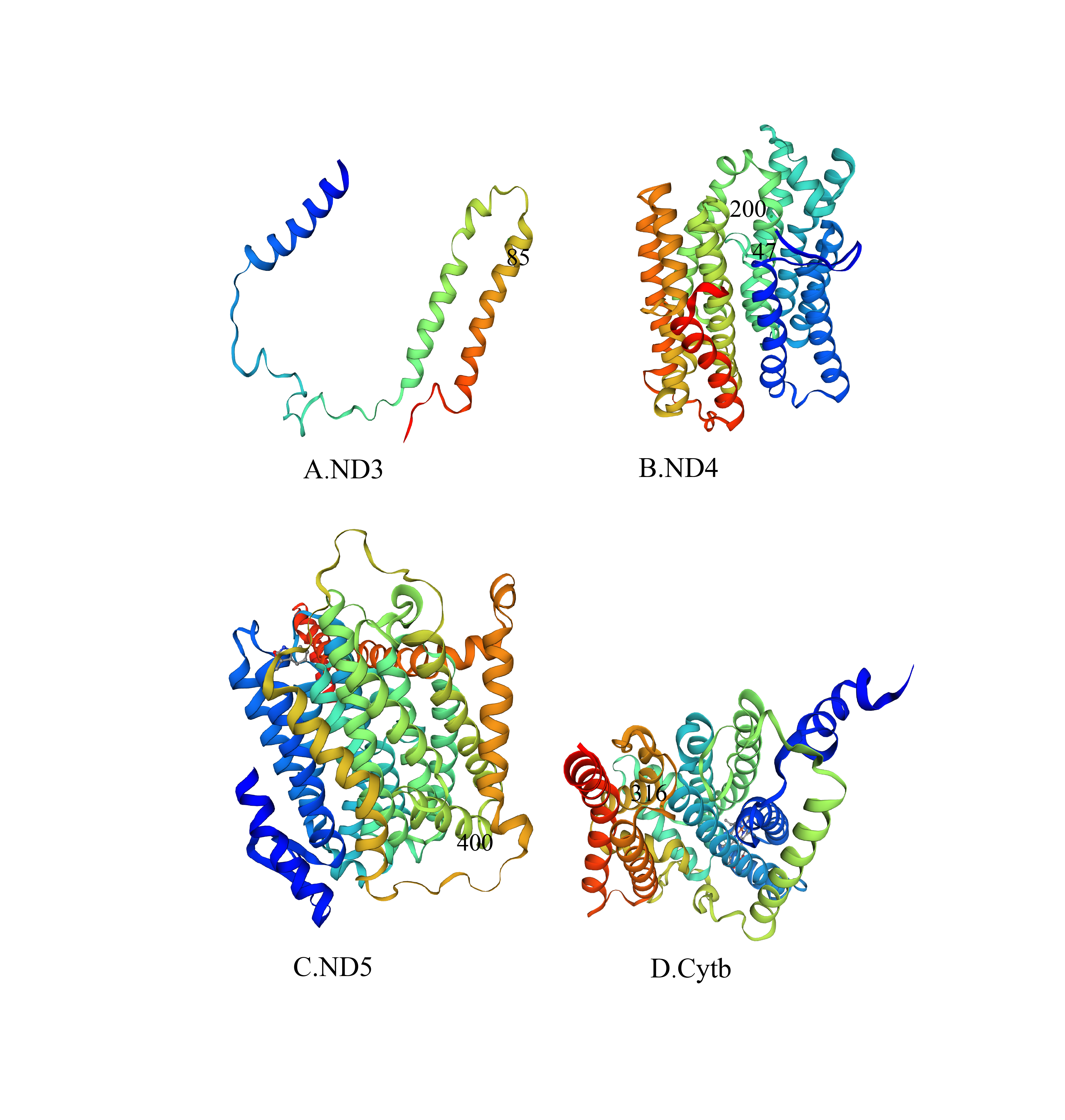

Supplement: Supplementary file 1 [file animals-13-01593-s001.zip › Figure S3 protein.png]
